# MEMS Reliability: On-Chip Testing for the Characterization of the Out-of-Plane Polysilicon Strength

**DOI:** 10.3390/mi14020443

**Published:** 2023-02-13

**Authors:** Tiago Vicentini Ferreira do Valle, Stefano Mariani, Aldo Ghisi, Biagio De Masi, Francesco Rizzini, Gabriele Gattere, Carlo Valzasina

**Affiliations:** 1 Department of Civil and Environmental Engineering, Politecnico di Milano, Piazza Leonardo da Vinci 32, 20133 Milano, Italy; 2 STMicroelectronics, via Tolomeo 1, 20010 Cornaredo, Italy

**Keywords:** MEMS stopper, polysilicon, brittle materials, tensile strength, fracture

## Abstract

Polycrystalline silicon is a brittle material, and its strength results are stochastically linked to microscale (or even nanoscale) defects, possibly dependent on the grain size and morphology. In this paper, we focus on the out-of-plane tensile strength of columnar polysilicon. The investigation has been carried out through a combination of a newly proposed setup for on-chip testing and finite element analyses to properly interpret the collected data. The experiments have aimed to provide a static loading to a stopper, exploiting electrostatic actuation to move a massive shuttle against it, up to failure. The failure mechanism observed in the tested devices has been captured by the numerical simulations. The data have been then interpreted by the Weibull theory for three different stopper sizes, leading to an estimation of the reference out-of-plane strength of polysilicon on the order of 2.8–3.0 GPa, in line with other results available in the literature.

## 1. Introduction

Stoppers act by constraining the motion of movable masses. For micro electro-mechanical systems (MEMS), such a motion can be due to mishandling and is therefore characterized by large uncertainties at the design stage. Due to MEMS geometry and working conditions, reliability issues can be linked to either in-plane or out-of-plane motions. This distinction is important for MEMS structures, since the microfabrication process adopted to build the movable parts provides a columnar morphology to the relevant polysilicon films, see e.g., [1,2]. As movable structures and stoppers are grown together, they basically inherit the same reliability issues in relation to the film morphology. With its shape and dimensions, each single stopper plays an important role during the whole life cycle of MEMS devices, since it assures that excessive motions do not take place to cause failure of flexible parts [3], also accounting for stiction [4]. Industrial tests carried out on MEMS primarily aim at proving the survivability of the whole device; only as an ancillary result, the stopper behavior was sometimes investigated [5]. This approach has led to very few systematic studies having as objective the quantification of the stopper resistance against repeated actions such as impacts. The corresponding load-carrying capacity is of primary importance, as a failure of small parts in the cavity hosting the movable structure can lead to short circuits or can constrain the device motion if debris becomes entrapped in the small gaps between the movable masses and the surrounding stators.

In this work, an on-chip testing device is proposed to investigate the load-carrying capacity of stoppers used to constrain the in-plane motion of movable parts. The obtained results could then be adopted to assess the out-of-plane strength of an epitaxially grown polysilicon film, independently of whether it is the structural material of stators or rotors. Since an electrostatic actuation and a capacitive sensing have been chosen in a quasi-static framework, the forces applied to the stoppers resulted to be relatively limited and, therefore, the stoppers were designed to display a small footprint. On the other hand, the test setup has allowed a clear quantification of the failure force and, as a consequence, of the material strength. Three different stopper sizes have been considered, by changing one of the in-plane dimensions to also provide insights on size effects related to the failure of the brittle polysilicon. In relation to the specific reliability issue here addressed, once the electrostatic force leading to stopper failure has been determined, it is possible to infer the polysilicon strength provided that the stress state is correctly evaluated in the reference volume where failure occurs. This is implicitly related to the brittleness of polysilicon, so that the maximum principal (tensile) stress at failure is assumed to match its tensile strength according to a Rankine-like criterion [6].

Hence, the tests to determine the stopper carrying capacity could be exploited to assess the out-of-plane polysilicon strength. As shown in the following, since the direction of the maximum principal stress is aligned with the direction of the columnar growth of the silicon grains (i.e., with the out-of-plane direction), a relatively understudied failure condition has been considered. In fact, in the majority of the configurations tested in the past, crack propagation occurred with a front almost aligned with the growth direction; see for example [7,8]. This somehow depends on the relative importance of reliability issues related to stoppers, in comparison with those of other mechanical parts judged critical for the MEMS structure, such as suspension springs. Regarding the assessment of the stopper bearing capacity, a systematical analysis has never been reported; the newly collected experimental data have been then compared with results of numerical simulations to mutually assess their accuracy, rather than considering available data related to other failure mechanisms.

As for all the brittle materials, polysilicon strength is significantly dependent on the flaws induced by the manufacturing process, which are typically located close to the external surface of the films [9]. The present work deals with polysilicon films made with the ThELMA (Thick Epipoly Layer for Microactuators and Accelerometers) process [2]; for other processes, interested readers can refer to the review [7,10,11,12]. Published results on 15 μm-thick polysilicon films reported a strength of about 4 GPa for the considered process, as obtained via single-edge notched tensile tests carried out with on-chip testing devices [2]. Such results were characterized by a strength higher than that relevant to other manufacturing processes [10]. Additional studies on the same ThELMA 15 μm-thick polysilicon film reported also a toughness slightly higher than that of other polysilicon materials, amounting to about 1.3 MPa m^1/2^ vs. 0.84–1.24 MPa m^1/2^ [13,14]. It is also worth mentioning that a ThELMA 0.7 μm-thick polysilicon film showed a lower strength, characterized by a 1.82 GPa Weibull scale parameter, more comparable with other polysilicon film properties [15]. In all the mentioned cases, a Weibull approach was adopted to assess the results from the theoretical point of view, as is often done for brittle materials. For the ThELMA polysilicon, relatively high values of the Weibull shape parameter were obtained for the thick films, while lower values were observed for the thin ones [2,13]. As mentioned above, due to the difficulties in testing and to the focus on the most common types of failure of the films, the direction of the tensile stress has been typically oriented in the plane. While there is no hint of a different behavior, the testing configuration presented in this work allows assessment of the out-of-plane strength from a truly quantitative point of view.

The remainder of this work is arranged as follows. The experimental setup, including the proposed testing device, is described in Section 2. The modeling procedure, combining analytical formulae and numerical simulations, is next detailed in Section 3. Results of data reduction are collected and commented in Section 4. Finally, some conclusions and suggestions for future works are drawn in Section 5.

## 2. Experimental

**Figure 1:**
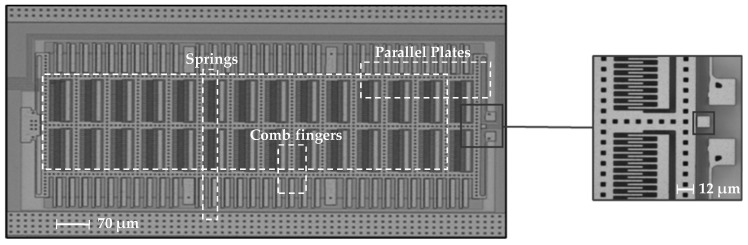
Micrograph of the device used in the experimental campaign; the inset on the right shows the stopper geometry.

The proposed testing device is shown in Figure 1. It consists of a massive shuttle equipped with 504 comb finger capacitors and 60 parallel plate capacitors, the latter distributed at the shuttle sides. Such a huge number of capacitors has been designed to allow the shuttle to move (horizontally to the right in Figure 1), touching the single stopper placed on one side, then pushing it with an electrostatic force able to cause its failure. The shuttle is connected to the substrate by beams located close to its corners. In the position at rest, the shuttle and the stopper have a target gap of 1.4 µm in between, see again Figure 1.

The device has been fabricated via the ThELMA process and shows a film thickness of 24 μm. Its footprint is about 1080 μm × 490 μm. The epitaxial growth of the structural polysilicon layer has been obtained over a 2.5 μm–thick thermal oxide layer and a plasma-enhanced chemical vapor deposited 1.6 μm–thick layer. After the epitaxial growth, a deep reactive ion etching stage has been adopted to dig trenches and holes; subsequently, the sacrificial oxide has been removed, to allow releasing the suspended structure. Further details can be found in [2].

Three different in-plane stopper sizes have been considered. All the geometries are characterized by a cross-section at the bottom featuring a dimension of 3.9 µm in the direction parallel to the shuttle motion; the other dimension has been set to vary and take the values 3.9, 4.4 and 4.9 µm. The choice to vary only the in-plane size of the stopper perpendicular to the motion of the movable parts has been adopted to allow the moment of inertia of the failing cross-section connecting the stopper itself to the substrate to vary linearly with it. The other way around, by varying instead the dimension parallel to the motion, the aforementioned moment of inertia would consequently change cubically, impacting considerably the load-carrying capacity of the entire device.

The experimental setup consisted of a probe station with an optical microscope, micromanipulators, an Agilent E4980A capacitance meter (Agilent Technologies, Santa Clara, CA, USA), two Agilent 6614C digital DC power supplies (Agilent Technologies, Santa Clara, CA, USA), and one AVHzY RD6018 (HangZhou RuiDeng Technology Co., Ltd., Hangzhou, China) DC power supply. To assess the pristine condition of the stopper, the shuttle has been first pushed against the stopper itself by imposing a DC voltage to the comb finger stators, up to 40 V. This first stage has been carried out by means of the capacitance meter DC bias. In a second stage, an additional DC voltage has been applied to the parallel plate capacitors by exploiting the DC power suppliers connected in series. The latter voltage has been increased stepwise, 0.5 V each step, until the failure of the stopper. The capacitance meter connected to the comb fingers has provided the measure of the capacitance change ΔC induced by the voltage rise. Whenever stopper failure has occurred, a pull-in event caused by the contact of the shuttle and the parallel plate surfaces has been caught in real time in the ΔC−V curve; the said pull-in voltage has been next exploited to identify the failure condition. To make sure that failure has occurred as expected, a post-mortem testing has been performed with the capacitance meter only, to record a further ΔC−V response and compare it to the former one, acquired during the first two stages of the same test. The described experimental setup has achieved two important objectives: first, it has allowed to run a static test, with voltage increased smoothly to control the stress state in the failing part and also allow data reduction in a clear way [16]; second, it has granted to identify the conditions leading to stopper failure through a pull-in, partially removing the uncertainties at this length-scale.

To avoid a loss of structural integrity when the short circuit is formed because of the pull-in event, during the second stage of the test the current has been limited to a maximum value of 3 mA in the DC power supply chain. This empirically set value has been proven quite reliable, since no devices were burnt; however, some devices have lost their functionality after the test, even without stopper failure, which implied that the aforementioned value should be further reduced.

## 3. Numerical Modeling and Comparison with the Experimental Evidence

**Figure 2:**
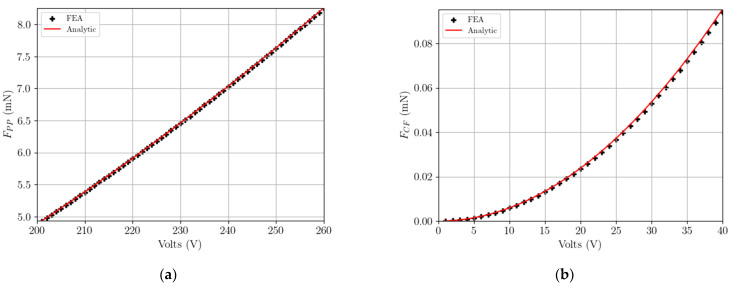
Comparison between analytical and numerical solutions, in terms of the electrostatic forces (**a**) $F_{PP}$ and (**b**) $F_{CF}$ as functions of the applied voltage.

To properly move from the acquired voltage at failure to the correspondent stress level, the relevant electrostatic forces have been evaluated. The electrostatic force in case of a parallel plate ($F_{PP}$) or a comb finger ($F_{CF}$) capacitor can be respectively estimated according to [9]. For a single parallel plate or comb finger capacitor they respectively read:

(1) $$F_{PP}^{*}=\frac{\epsilon Bl}{2}{\left(\frac{V_{PP}}{d_{PP}}\right)}^{2},$$

(2) $$F_{CF}^{*}=\frac{\epsilon B}{d_{CF}}\;V_{CF}^{2},$$

where: $V_{PP}$ is the voltage applied to the parallel plate capacitor; $V_{CF}$ is the voltage applied to the comb finger stator; ϵ is the dielectric permittivity of vacuum; B is the out-of-plane thickness of the polysilicon film; $d_{pp}$ is the gap between the plates; l is the facing dimension of the parallel plates; $d_{CF}$ is the gap between the movable finger and each stator. The results provided by the above analytical equations have been found to be in good agreement with those relevant to a numerical model of the entire device developed with Comsol Multiphysics [17]. By considering that in all the tested devices there are $N_{PP}=60$ and $N_{CF}=504$ parallel plate and comb finger capacitors, a comparison between the analytical and numerical solutions is depicted in Figure 2, in terms of the total attractive forces $F_{PP}=N_{PP}\;F_{PP}^{*}$ and $F_{CF}=N_{CF}\;F_{CF}^{*}$ as functions of the relevant values of voltages $V_{PP}$ and $V_{CF}$. The difference between the two solutions is shown to be negligible, on the order of 1% at most. Therefore, for the designed geometry fringe effects can be considered irrelevant.

**Figure 3:**
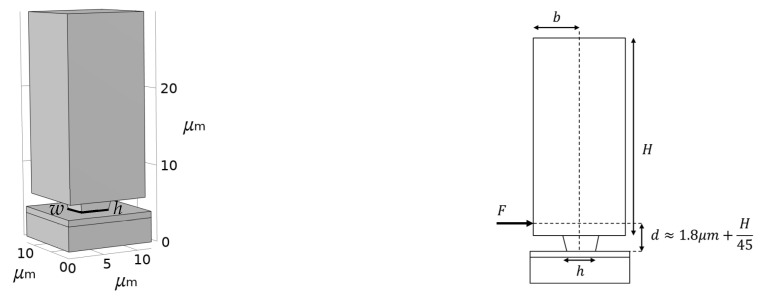
Stopper geometry and (distributed) contact force induced by the shuttle motion.

To move from the electrostatic force to the stress acting on the failing surface, a beam bending model has been considered, assuming the stopper to be a cantilever held fixed at its bottom cross-section connecting it to the substrate. Accordingly, the overall force transmitted from the shuttle to the stopper leads to a maximum tensile stress that reads:

(3) $$\sigma_{\;}=\frac{6\;F\;d}{w\;h^{2}},$$

where, see Figure 3: $F=F_{PP}+F_{CF}$ is the in-plane force applied to the stopper; w and h are, respectively, the width and the depth of the stopper; and d is distance between the line of action of the contact force and the substrate, as computed through the finite element (FE) simulation. The contact force displays also an out-of-plane component due to the bent geometry of the stopper; however, the numerical simulations have reported a value of this component to be three orders of magnitude smaller than the in-plane one and has been therefore disregarded in the present analysis. During the second stage of the tests, the voltage at the comb fingers has been held constant at $V_{CF}=40\;V$ while $V_{PP}$ has been increased. By monitoring the value of the voltage at the parallel plate capacitors till $V_{PP}=V_{R},$ when the pull-in occurs, the tensile stress at failure can be easily computed accordingly to Equation (3).

Such a solution does not account for the stress concentration induced by the actual stopper geometry reported in Figure 3, leading to an underestimation of the stress state in the process region. To assess the stress concentration factor (SCF), a FE analysis has been further performed. In it, a Coulomb friction coefficient equal to 0.3 has been adopted between the two polysilicon surfaces of shuttle and stopper coming into contact, see [18]. The electrostatic force $F_{R}$ leading to the failure of the stopper has been imposed and all the major geometrical details of the stopper itself have been considered to correctly determine the aforementioned SCF. In particular, the fillet radius for the horizontal and vertical edges in the (notched) failing region has been assumed equal to 0.6 μm, as suggested by a scanning electron microscope image of the stopper. To make sure that the results are mesh-independent, a series of simulations has been carried out by decreasing the mesh size, till convergence in the reported solution of the stress field in the failing volume. The finer mesh consisted of 717,057 quadratic tetrahedral elements, with the smallest edge size equal to 0.012 μm at the fillet corners.

**Figure 4:**
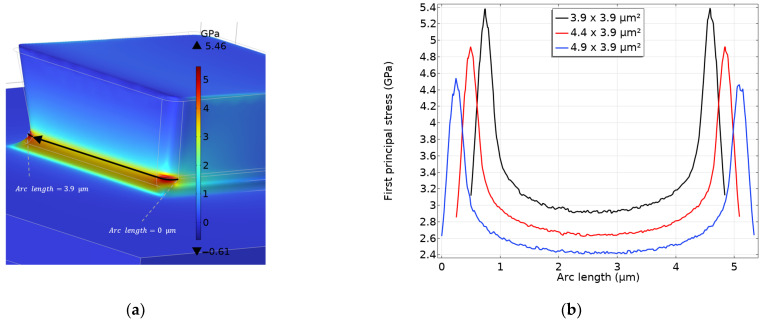
(**a**) Schematization of the path along the front surface of the stopper, where the stress profiles in (**b**) have been obtained with dedicated FE simulations for the three stopper geometries.

The resulting stress profiles along the path at the front surface of the stopper anchor shown in Figure 4a, are reported in the graph of Figure 4b for the three stopper sizes. The plots show a slight asymmetry due to the adopted mesh; it is anyhow possible to observe that the stress concentration is maximum close to the corners, while in the central part of the path the local SCF is roughly equal to 1.8 for all the geometries. The SCF, henceforth termed k, has been then computed by considering the mentioned peaks in the stress profiles, resulting to be k=3.37/3.43/3.55 if moving from the smallest to the largest geometries, with an average value of $\bar{k}=3.45$. As the behavior of polysilicon has been assumed brittle, the elastic solutions discussed in what precedes are valid for any value of the applied voltage and can therefore be adopted to scale the solution computed via beam bending.

**Figure 5:**
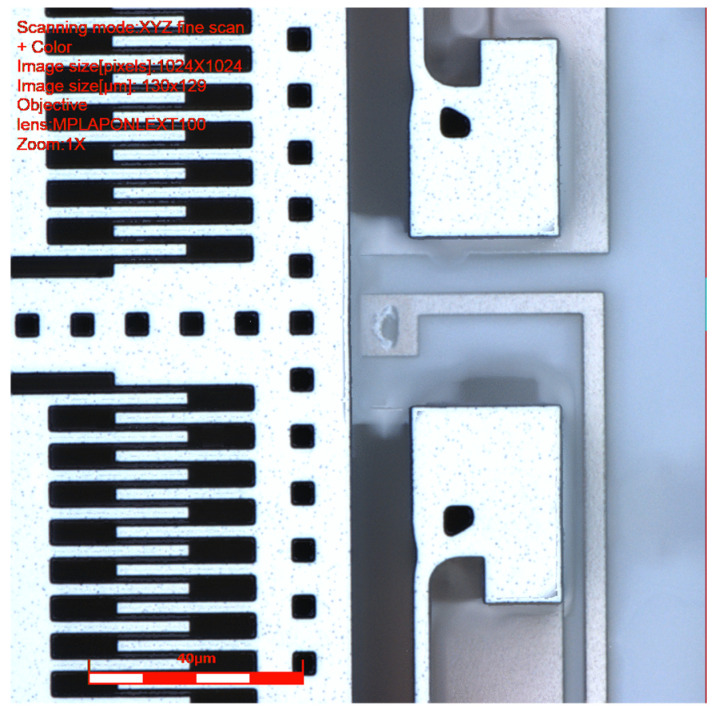
Confocal microscopy image of a broken stopper.

A third series of analyses has then been adopted to assess the consistency of the numerical solutions also in terms of the morphology of the fracture surface, which has been observed in the experiments such as in Figure 5. Purely mechanical analyses have been carried out at the stopper level by using a smeared crack approach to model the tensile stress-induced failure of the brittle polysilicon [19]. Readers can find in [19] a discussion on the use of such a model for polysilicon, and on the relevant implementation in a commercial software [20]. For the purpose of the present discussion, it is worth mentioning that a mode I (opening) fracture is incepted when the material tensile strength is attained; later, provided that continuous loading is guaranteed, in the process zone ahead of the crack front an inelastic (dissipative) contribution is added to catch strength reduction (softening) as a function of crack opening. Alternative approaches based on a cohesive description of the damaging region can provide more robust results, see e.g., [21], but do not add additional insights in terms of the morphology of the crack surface. In fact, according to the procedure adopted in [19], linking the crack strain to a corresponding displacement jump through a characteristic length of the material, strain localization can be limited, and results can become (almost) mesh-insensitive, if the element size is small enough to accurately resolve the stress state.

Since the stopper is connected to the substrate through a silicon dioxide layer, mechanical properties have to be provided for both (poly)silicon and the aforementioned dioxide. In the analyses, the properties for the materials have been set as reported in Table 1, see also [14,21]. Since the film is actually a polycrystalline material, the values here collected have to be considered as effective, or averaged at the level of grain aggregate, on its own to be considered as large enough to allow modelling the structural film as in-plane homogeneous. The reported values of fracture toughness refer instead to the actual out-of-plane failure mechanism, involving a crack surface which is parallel to the substrate.

**Figure 6:**
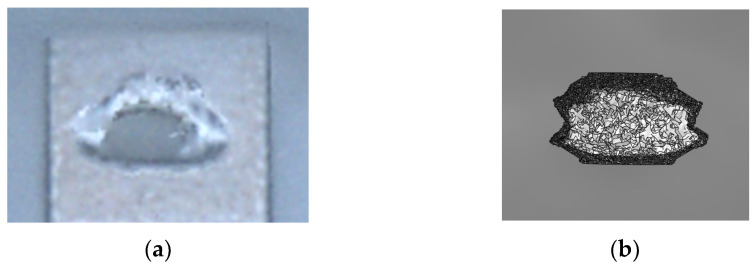
(**a**) Detail of Figure 6 showing a top view of the experimental fracture profile, and (**b**) result of the corresponding FE simulation.

As reported in Figure 6, the experimental and numerical fracture surfaces have agreed quite well, testifying that the attained conditions for stopper failure at its bottom are in accordance with the real phenomenon. To also assess whether repeated impacts during testing can give rise to the same fracture profile observed experimentally, the same FE model has been adopted under a dynamical contact load. The final fracture surface has resulted not to differ from the static case, suggesting that the failure mechanism induced by an accidental impact could be similar to that induced by quasi-static loading.

## 4. Statistical Analysis of the Results

**Figure 7:**
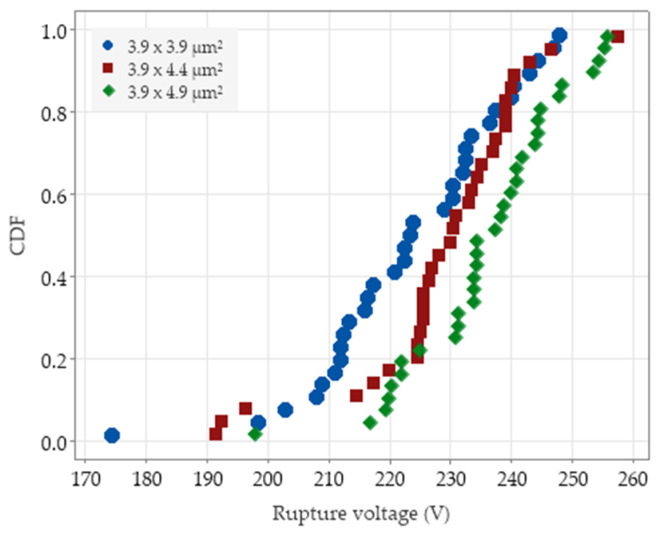
Cumulative distribution functions of the voltage at stopper failure, for the three considered sizes reported in the legend.

Using the test setup described in the Section 2, 33 tests have been carried out for each stopper size. Figure 7 shows the resulting cumulative distribution function (CDF) of the failure voltage for each stopper size: it can be seen that the three stopper sizes resulted in (slightly) different probability curves.

To interpret the results, a two-parameter Weibull statistic has been adopted [2,9,22,23]. The CDF of the failure probability $P_{f}$ can be then written as [7]:

(4) $$P_{f}=1-\exp\left[-\frac{1}{A_{0}}\int_{A_{0}}^{\;}{\left(\frac{\sigma \left(x\right)}{\sigma_{0}}\right)}^{m\;}dA\right]=1-\exp\left[-{\left(\frac{\sigma_{f}}{\sigma_{0}}\right)}^{m\;}\frac{1}{A_{0}}\int_{A_{0}}^{\;}g^{m}\left(x\right)\;dA\right],$$

where: $\sigma_{f}$ is the tensile failure stress; $\sigma_{0}$ is a scale parameter, namely the stress per unit area corresponding to 63.2% of the test failures in the probability curve; m is the shape parameter, also known as Weibull modulus, which measures the dispersion of the results (namely as m grows, the distribution becomes narrower); $A_{0}$ is the surface area wherein flaws do affect failure, also known as the representative area [24]; $g\left(x\right)$ is the function expressing the normalized stress distribution in the $A_{0}$ area, x being the position vector.

The choice of this two-parameter Weibull distribution in place of a three-parameter one, which also includes a threshold stress [9,10,22], has been suggested by the relatively limited data availability for every stopper size, see also [10,25], even if its applicability is still controversial [10,24].

Three alternative approaches have been then adopted for data reduction, labeled in the following as A, B, and C. With approach A, a uniform stress distribution, as computed with Equation (3) corrected via the SCF k, has been considered. As clearly visible in Figure 4b, the stress distribution in the failing region is far from being uniform; therefore, such an approach has only to be considered a naïve trial to simplify as much as possible the data reduction procedure. Under such a uniform stress assumption, it turns out that $g\left(x\right)=1$ in Equation (4). By assuming the Weibull parameters m and $\sigma_{0}$ to be material-dependent, for the three stopper sizes the failure probability simplifies to:

(5) $$P_{fj}=1-\exp\left[-\frac{A_{j}}{A_{0}}{\left(\frac{\sigma_{f}}{\sigma_{0}}\right)}^{m\;}\right]\;\;\;\;j=1,2,3,$$

where $A_{j}$ is the area affected by the failure process for the j-th size.

To estimate m and $\sigma_{0}$ from the collected experimental data, the maximum likelihood approach has been adopted, as described in the appendix of [24]. By considering the logarithm of the product of all the data point likelihood, i.e., $\ln\frac{dP_{f}}{d\sigma_{f}}$ for each size, and by taking its partial derivatives with respect to the $m,\;A_{0}$ and $\sigma_{0}$, two independent equations are obtained. By allowing for all the stopper sizes, the aforementioned equations become:

(6) $$A_{0}\sigma_{0}^{m}=\frac{1}{33\cdot 3}\sum_{j=1}^{3}\sum_{i=1}^{33}A_{j}\sigma_{fij}^{m},$$

(7) $$\left(\sum_{j=1}^{3}\sum_{i=1}^{33}A_{j}\sigma_{fij}^{m}\right)\left(\frac{33\cdot 3}{m}+\sum_{j=1}^{k}\sum_{i=1}^{n_{j}}\ln\sigma_{fij}\right)-33\cdot 3\cdot \sum_{j=1}^{3}\sum_{i=1}^{33}A_{j}\;\sigma_{ij}^{m}\ln\sigma_{fij}=0,$$

where: 33 is the (already noted) number of data points collected for each stopper size; 33·3=99 is thus the total number of data points provided by the experimental campaign; $\sigma_{fij}$ represents the i–th experimental datum relevant to the j–th stopper size. By setting $A_{0}$ as the smallest failing surface among the specimen sizes, it has been possible to solve the nonlinear Equation (7) for m, e.g., through a Newton–Raphson procedure. Equation (6) has been next used to determine $\sigma_{0}$. This identification procedure has led to the following estimated values: m = 8.45 and $\sigma_{0}$ = 4.46 GPa.

According to the Weibull approach for brittle materials, the survival probability of the whole device is the joint probability of survival of all its elementary parts. Hence, larger volumes of materials effectively stressed tend to exhibit a lower tensile strength, because of the higher probability of finding a defect inside them [26]. In another study on polysilicon made through the same production process [2], m was estimated to be 6.67 due to the dispersed experimental data. The value for the shape parameter here reported testifies the less scattered results, and such a range is not unusual: in the ensemble of data collected in [26], depending on the geometry and loading profiles, m was reported to range between 5 and 14.

By performing a goodness-of-fit test, such as the Anderson–Darling (AD) test [27], it has resulted that a normal distribution could better fit the data, since AD = 0.72 for the normal distribution and AD = 1.01 for the Weibull distribution. The mean of the Weibull distribution for all the data points, given by $\bar{\sigma }=\Gamma \left(1+\frac{1}{m}\right)\sigma_{0}=4.92$ GPa [2], has resulted to be very close to the mean of the normal distribution, which reads instead 4.93 GPa. This effect could be explained by some additional factors influencing the results at the microscale, such as the over-etch and instrumentation uncertainties, see also [2,28,29]. These errors were not accounted for in data reduction, even if they could affect the probability distributions fitting the data. An alternative explanation, given in [30], pointed to the relationship between the stressed region and the representative volume: when the former is comparable in size to the latter, the failure CDF appears as a normal distribution in the core with Weibullian tails. The mentioned representative volume dimensions are linked to the spacing between the strength-limiting flaws that in fact establish an intrinsic length-scale for the material. With this latter interpretation, the intrinsic length would be on the order of the size of the regions affected by stress concentration at the re-entrant corners, which is about a few hundreds of nm; see also the width of the spikes in the stress plot of Figure 4. A better understanding of this issue, however, would require an investigation of the surface flaws, e.g., by means of a scanning electron microscope, which is outside the scope of this paper.

**Figure 8:**
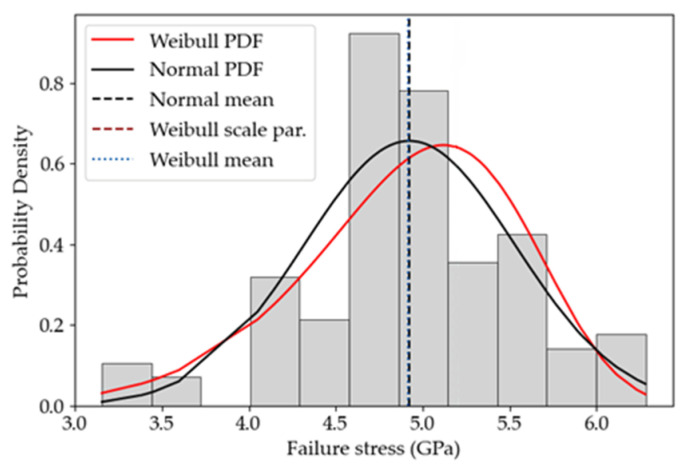
Data reduction approach A. Histogram of the failure stress of all the tested devices, and fitting with two-parameter Weibull and normal probability distributions. The mean values relevant to the two distributions are represented by the vertical dotted lines.

**Figure 9:**
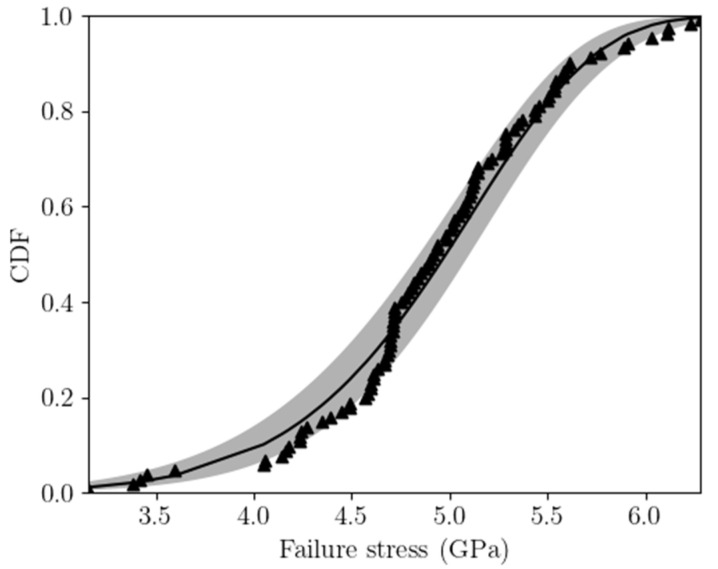
Two-parameter Weibull CDF for the whole tensile strength data set. The shaded area in the plot represents the 95% confidence interval.

If the whole set of data for the three geometries is considered without any correction factor to account for the different sizes, see Figure 8, a mean value of 4.92 GPa and a standard deviation of 0.61 GPa have been obtained to characterize the statistics of the out-of-plane tensile strength. This mean value looks higher than others previously reported in the literature, including also polysilicon films made with the same microfabrication process [2,26,31,32], but those results referred to an in-plane failure mode. The corresponding Weibull CDF reported in Figure 9 helps visualize the range of computed failure stresses; even though the two-parameter Weibull approach does not allow for a stress threshold value, from this plot it is possible to choose a survival probability $1-P_{f}$ and to estimate the relevant value of the maximum allowed design stress.

Moving now to the data reduction approaches B and C, the stress distribution over the failing region (see Figure 4a), as computed via the numerical simulations described in Section 2, has been fully considered. The procedure to infer the values of m and $\sigma_{0}$ is the same adopted before via Equations (6) and (7), but the integral in Equation (4) has now to be computed. According to [24], the idea is to find the equivalent size for each stopper size that would provide the same strength distribution of the non-uniform case; for this purpose, it is necessary to compute the integral according to:

(8) $$P_{fj}=1-\exp\left[-\frac{1}{A_{0}}\int_{A_{0}}^{\;}{\left(\frac{\sigma \left(x\right)}{\sigma_{0}}\right)}^{m\;}dA\right]=1-\exp\left[-{\left(\frac{\sigma_{f}}{\sigma_{0}}\right)}^{m\;}\frac{1}{A_{0}}\int_{A_{0}}^{\;}g^{m}\left(x\right)\;dA\right]\;=1-\exp\left[-\frac{A_{j\;}\varphi_{j\;}}{A_{0}}{\left(\frac{\sigma_{f}}{\sigma_{0}}\right)}^{m}\right]$$

wherein the adimensional coefficient $\varphi_{j}$, implicitly defined in Equation (8), allows to scale $A_{j}$ to define an effective area affected by a failure process. In this equation, $\sigma_{f}$ represents the maximum value of the stress over the failing region. As before, by first setting $A_{0}$ the procedure to tune m and $\sigma_{0}$ is ruled by Equations (6) and (7). The integral of $g\left(x\right)$ in Equation (8) has been computed by considering the stress distribution depicted in Figure 4b, properly scaled [7], by exploiting the results of the FE simulations.

The choice of $A_{0}$ in Equation (7) obviously makes a difference. As it represents the area of the weakest elementary part of the material leading to failure in the Weibull representation, it can be related to the dimension of the critical flaws. Since its setting from the raw data is not trivial [24], it is often taken equal to the area of the most stressed region, wherein failure is triggered with the highest probability. In our analysis the stress field is not uniform, see again Figure 4, which complicates the matter; further than this, it was pointed out in [24] that at small length-scales the effective area could be size-dependent too and also geometry-dependent. In [25] it was suggested that, in order to properly assess size effects within the context of Weibull interpretation for brittle materials, specimens with sizes spanning 2–3 different orders of magnitude need to be used; in the present investigation, all the sizes are comparable and, accordingly, the Weibull parameters obtained with data reductions relevant to the three stopper sizes have been adopted to check the consistency of each approach.

**Figure 10:**
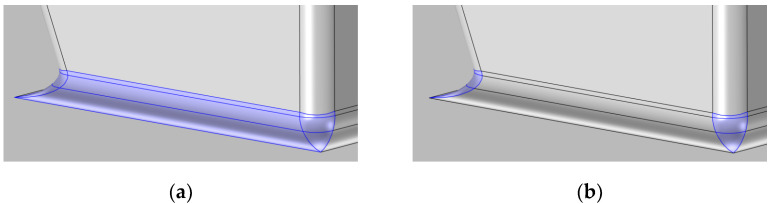
$A_{0}$ regions highlighted, as adopted for the computation of the scale parameter $\sigma_{0}$: (**a**) frontal fillet and corners; (**b**) two corners only.

To investigate the issues related to the setting of $A_{0}$, the two alternatives shown in Figure 10 are considered in the following: $A_{0}$ consisting in the fillet area in Figure 10a (approach B); $A_{0}$ consisting in the reentrant corners only, where stress intensification occurs as shown in Figure 10b (approach C).

By adopting the approach B, the stress parameter has resulted to be $\sigma_{0}\;$= 2.80 GPa, and m = 8.50. The $\sigma_{0}$ value becomes lower than the one obtained with the approach A, and is also more similar to those reported in the literature [2,7]. According to this data reduction approach, it can be claimed that the stress concentration regions at the corners of the stopper, leading to the peaks in the function $g\left(x\right)$ shown in Figure 4, are those actually leading to the inception of the entire failure process. The aforementioned discussion related to an intrinsic length as the size of the volume where stress concentration at the re-entrant corner occurs, would then still hold true.

In view of the results of approach B, approach C has explored the hypothesis that a stress level beyond a critical threshold for polysilicon has led to failure starting at the reentrant corners only. Here, if a critical flaw is present due to intrinsic material properties and to the manufacturing process, the event can be triggered. With such an analysis of the raw data, the parameters have turned out be $\sigma_{0}$ = 2.98 GPa, and m = 9.0.

By comparing the values of $\sigma_{0}$ estimated through the three approaches, it appears that approach A stemming from the rough approximation $g\left(x\right)=1$, which has actually allowed to avoid a fine post-processing of the FE analyses, has led to a higher value of $\sigma_{0}$. This is due to the fact that a uniform stress profile has been assumed in the cracking region, without accounting for the stress amplification induced by the corners and by the specific stopper geometry. Approach B has given instead the lowest $\sigma_{0}$ value, due to the fact that the experimental data have been interpreted by assuming that failure is triggered in a region which is probably bigger that the real one, including the entire fillet loaded in tension at the frontal surface of the stopper. In this way, the SCF has been smeared out in this region, which has played a critical role in the entire failure process after its triggering at the corners. Hence, the $\sigma_{0}$ value given by approach C has been higher than the one provided by approach B, since the non-uniform stress distribution around the peaks at the corners has been accounted for in smaller representative areas. To discriminate between the results of approaches B and C, the histogram of the probability density function in Figure 8 can be also accounted for. Experimental data seem to display a bimodal distribution; one could then argue that it would be necessary to include the whole fillet region in the analysis, because flaws of different sizes could lead to failure in correspondence of two different stress levels, and this could happen either in the region corresponding to the plateau of Figure 4b or in the region where the stress peaks occur. Conversely, a unimodal distribution has been adopted to interpret the data statistics; therefore, it has been assumed that a unique, dominant flaw is leading to failure, and this dominant flaw has to be located where the stress peaks are located. Accordingly, to make everything consistent, the results obtained with approach C are to be preferred for further analyses in future activities.

## 5. Conclusions

A new MEMS testing device has been designed to assess the load-carrying capacity of small-sized stoppers attached to the die substrate and, therefore, to characterize the out-of-plane strength of columnar polysilicon films. The main findings of the present work can be listed as follows.

- The scattered experimental results have been interpreted within the frame of Weibull statistics, to identify the stress level corresponding to a certain probability of failure, accounting for three different tested sizes of the stoppers.
- A discussion about three alternative interpretations of the experimental data has been provided, to either allow or not for the non-uniform stress distribution in the critical region where the failure event is going to be triggered.
- The two more reliable approaches have provided an estimation of the characteristic tensile strength of polysilicon, actually of the scale parameters of the Weibull distribution best fitting the experimental data, on the order of 2.8–3.0 GPa, which is quite in line with data available in the literature for similar structural films, even if tested in different modes.
- The area where flaws can actually affect failure seems to be focused on the fillet region close to the edge of the stopper, at least for the considered stopper geometry.

The parameters of the Weibull distribution fitting the data could next be adopted to design reliable MEMS stoppers of similar size, by accepting a certain failure probability according to the rationale here discussed for brittle materials. For MEMS of considerable larger sizes, a deeper investigation is needed. The proposed testing procedure, which has the merit of an easy setting of the electrostatic actuation, can be scaled up to larger forces and stopper geometries with some difficulties, due to the voltage values adopted in the testing phase.

Numerical simulations here reported have also suggested that the stopper failure in case of mishandling or repeated impacts, might coincide with the quasi-static one. A complete stopper failure could be reasonably avoided in view of the high values of the voltage used for the characterization. Nevertheless, in cases characterized by a high probability of survival and corresponding design stress level, the creation of chips or small debris remains an issue needing additional investigation.

## Figures and Tables

**Table 1:** Materials properties adopted in the crack propagation simulations.

Material	Young’s Modulus (GPa)	Poisson’s Ratio (-)	Fracture Energy (J/m^2^)
Polysilicon	150	0.22	7
Silicon dioxide	70	0.17	7

## Data Availability

The data generated during and/or analysed during the current study are available from the corresponding author on reasonable request.
